# Combining genetic markers with stable isotopes in otoliths reveals complexity in the stock structure of Atlantic bluefin tuna (*Thunnus thynnus*)

**DOI:** 10.1038/s41598-020-71355-6

**Published:** 2020-09-07

**Authors:** Deirdre Brophy, Naiara Rodríguez-Ezpeleta, Igaratza Fraile, Haritz Arrizabalaga

**Affiliations:** 1 Marine and Freshwater Research Centre, Galway Mayo Institute of Technology, Dublin road, Galway, H91 T8NW Ireland; 2 Marine Research Division, AZTI, Txatxarramendi Ugartea Z/G, 48395 Sukarrieta, Bizkaia Spain

**Keywords:** Ecology, Genetics, Biogeochemistry

## Abstract

Atlantic bluefin tuna (*Thunnus thynnus*) from the two main spawning populations in the Mediterranean and Gulf of Mexico occur together in the western, central and eastern Atlantic. Stock composition of catches from mixing areas is uncertain, presenting a major challenge to the sustainable management of the fisheries. This study combines genetic and chemical markers to develop an integrated method of population assignment. Stable isotope signatures (δ^13^C and δ^18^O) in the otolith core of adults from the two main spawning populations (adult baselines) showed less overlap than those of yearlings (12–18 months old) from western and eastern nursery areas suggesting that some exchange occurs towards the end of the yearling phase. The integrated model combined δ^18^O with four genetic markers (SNPs) to distinguish the adult baselines with greater accuracy than chemical or genetic markers alone. When used to assign individuals from the mixing areas to their population of origin, the integrated model resolved some (but not all) discrepancies between the chemistry and genetic methods. Some individuals in the mixing area had otolith δ^18^O values and genetic profiles which when taken together, were not representative of either population. These fish may originate from another Atlantic spawning area or may represent population contingents that move away from the main spawning areas during the first year of life. This complexity in stock structure is not captured by the current two-stock model.

## Introduction

Increasing global food demands, climate change and habitat loss place unprecedented pressure on the world’s fish populations^1–3^. Declines in abundance signal an urgent need for reductions to exploitation levels while recoveries of some populations demonstrate the role of accurate assessment and effective management in reversing stock collapse^4,5^. A fundamental step in the effective management of a fishery is to identify the appropriate management unit (stock)^6^. This is unfortunately wrought with difficulties; fish populations rarely maintain geographically discrete distributions throughout their life cycle and many fisheries exploit mixed aggregations^7^. Populations that are sufficiently isolated during reproduction are genetically discrete and can be definitively distinguished using genetic markers^8^. However, populations that have recently diverged may not show detectable variation in selectively neutral genetic markers^9,10^. In addition, low levels of gene flow between neighbouring populations may preclude genetic discreteness while being insufficient to ensure replenishment of one by the other in the event of stock collapse^11,12^. A single stock may contain multiple biologically relevant units that differ in life history traits or behaviour with varying degrees of mixing between them^13,14^. Fisheries management must ensure that stock definitions used in assessment correspond to biologically relevant units. A flexible approach to stock identification is needed, which recognises the broad spectrum of stock structure scenarios that exist^7,15^.

Increasingly, both genotypic and phenotypic traits are being used in combination to characterise fish populations^16–19^. While genotypic differences definitively confirm that populations are reproductively isolated from each other, phenotypic variation can arise when groups of fish inhabit different environments for a substantial part of the life cycle and may not necessarily have a genetic basis. If multiple aggregations of spawning adults can be distinguished based on phenotypic traits, this indicates that they are to a large extent separate reproductive units. While occasional reproductive exchange may prevent genetic differentiation, at the temporal scale that is relevant for management the units are separate and should be managed accordingly^20^. Combining multiple markers can increase resolving power in stock discrimination and provide a more nuanced picture of population structure over various temporal and spatial scales^19,21,22^. The various genotypic and phenotypic markers provide different information about when and to what extent components in a population diverge. A particular challenge lies in combining the different types of categorical and continuous data that are generated by the various methods as well as incorporating technique-specific limitations into the interpretation.

The Atlantic bluefin tuna (*Thunnus thynnus*) (ABFT) is a highly migratory species that is widely distributed across the Atlantic ocean, from east to west^23^. The species’ high commercial value has led to intense exploitation, precipitating pronounced declines in abundance from the 2000’s and leading to its classification as a globally endangered species by the International Union for Conservation of Nature (IUCN)^24,25^. While the introduction of more restrictive management regulations has led to improvements in stock status, uncertainties remain in the assessment of ABFT^26–28^.

The International Commission for the Conservation of Atlantic Tunas (ICCAT) currently manages ABFT as two distinct stocks separated by the 45°W meridian: the western stock that spawns in the Gulf of Mexico and the eastern stock that spawns in the Mediterranean^23^. Tagging and otolith chemistry studies show that individuals from the eastern and western stocks undertake trans-Atlantic migrations and there is extensive overlap in the distribution of the two stocks^29–32^. Evidence from genetics^33,34^ and otolith chemistry^29,35^ supports natal homing of ABFT while tagged individuals show strong fidelity to spawning grounds^32^. However, the occurrence of mature fish^31,32,36,37^ and early larvae^38–40^ outside of the main spawning areas during spawning time suggests that the current two stock model may over-simplify ABFT stock structure. The limitations of the current management approach are recognised and alternative assessment models are being tested within a management strategy evaluation framework that incorporates different scenarios of population mixing^41,42^. To inform the development of plausible operating models for MSE, a method is needed to discriminate between the two stocks and to estimate mixing rates where their distributions overlap. The potential contribution of ABFT from other spawning areas also needs to be addressed through development and refinement of stock identification approaches.

Various genotypic^33,34,43–45^ and phenotypic^35,46–48^ population markers have been used to distinguish between ABFT from the eastern and western Atlantic. Of these, genetic markers (Single nucleotide polymorphisms: SNPs^34,45^) and otolith core stable isotope signatures^29,35^ are the most useful for assigning individuals to their natal areas. However, there is a degree of uncertainty associated with each method of population assignment. Estimates of mixing rates in the Central Atlantic and in the Eastern Atlantic off the coast of Africa vary widely between years and across studies that employ different methodological approaches^29,34,45,49^. Combining information from multiple population markers may help to reduce the uncertainty associated with each method individually. The aim of this study is to combine information from genetics and otolith stable isotopes to develop an integrated method of population assignment for ABFT. Population assignments obtained using one type of marker are compared and contrasted with those from the integrated method and the accuracy of each approach is evaluated. Various scenarios of bluefin mixing and population movements are considered in relation to the information provided by each method, together and in combination.

## Methods

### Sampling

**Figure 1:**
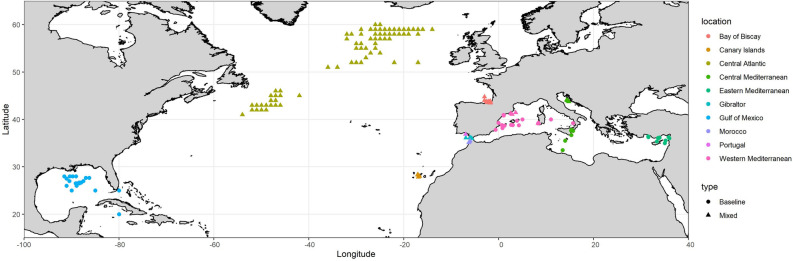
Map showing the sampling locations for adult Atlantic bluefin tuna in the baseline (collected during the spawning season: circles) and mixed (collected outside of the spawning season: triangles) samples. Colours indicate the locations referred to in the text. Map was created in R using the ggplot2 package version 3.2.1 URL: https://ggplot2.tidyverse.org^79^.

All samples were obtained under the provision of the ICCAT Atlantic Wide Research Program for Bluefin Tuna (GBYP) or from NOAA sampling programs. In the central north Atlantic, east Atlantic and Mediterranean Sea, fish were collected during commercial fishing operations using a combination of capture methods (traps, long-lining, bait boats, purse seine). Samples from the west Atlantic were collected from commercially caught ABFT (long line). The baseline samples used to characterise the Gulf of Mexico (GoM) and Mediterranean (Med) spawning populations consisted of mature adults (Med: > 135 cm, GoM: > 185 cm)^50,51^ collected from the main spawning areas in the GoM and the Med during the spawning period (GoM: April–June, Med: May–June)^23,52,53^ between 2009 and 2016. A total of 384 fish were included in the adult baselines (94 from the GoM and 290 from the Med). All of these fish were included in the analysis of otolith core stable isotopes, and 150 were analysed using both stable isotope and genetic methods (Table 1, Fig. 1). The genetic analysis was conducted as part of a previous study; the methodology is described fully by Rodríguez-Ezpeleta^34^ and is summarised here.

**Table 1:** Numbers and sizes of individuals used to characterise the Atlantic bluefin tuna  spawning populations in the Gulf of Mexico and Mediterranean Sea based on otolith core stable isotope signatures (adult baseline: chemistry) and stable isotopes combined with genetics (adult baseline: integrated).

	Length (cm)	Sample size								
	Mean (range)	2009	2010	2011	2012	2013	2014	2015	2016	Total
**Adult baseline: chemistry**										
Gulf of Mexico	247 (199–288)	44	15		11	11	13			94
Mediterranean	221 (138–282)		12	131	65	1		51	30	290
**Adult baseline: combined**										
Gulf of Mexico	241 (199–281)		15		8	10	12			45
Mediterranean	222 (170–272)				72	1		32		105

The mixed sample consisted of juvenile and adult ABFT of unknown spawning origin collected outside of the spawning season at various locations in the central and eastern Atlantic, and the western, central and eastern Mediterranean Sea (Table 2, Fig. 1). A total of 2,081 fish were analysed for otolith core stable isotopes. Of these, 750 were previously analysed using genetic markers^34^. An important objective of the research programme through which the data were generated was to address uncertainty related to stock mixing. Therefore, a high proportion of the samples were collected from areas for which estimated rates of mixing varied annually or between different methods^29,34,45,49^ (Central Atlantic, Portugal, Morroco and Canary Islands) with fewer samples from the Mediterranean Sea where the fishery for the eastern stock of ABFT is concentrated.

**Table 2:** Numbers and sizes of individuals in the mixed sample of juvenile and non-spawning adult Atlantic bluefin tuna analysed using otolith chemistry and genetic markers.

	Mixed sample									
	Length (cm) Mean (range)	Sample sizes Chemistry (genetics and chemistry)								
		2009	2010	2011	2012	2013	2014	2015	2016	Total
**Central North Atlantic**										
Central Atlantic (CA)	204 (121–275)	3 (0)	115 (0)	94 (45)	167 (73)	118 (49)	177 (76)	108 (57)		782 (300)
**East Atlantic**										
Bay of Biscay (BB)	100 (77–106)	104 (0)	108 (0)	104 (82)	52 (10)					368 (92)
Canary Islands (CI)	229 (206–263)					23 (23)	38 (0)	23 (0)	44 (42)	128 (65)
Morocco (MO)	145 (42–275)			33 (0)	49 (49)	58 (58)	49 (48)	50 (46)	50 (49)	289 (250)
Gibraltar (GI)	153 (51–239)		19 (0)	81 (0)						100 (0)
Portugal (PO)	209 (170–281)			93 (27)	30 (16)					123 (43)
**Western Mediterranean**										
Balearic Islands (BA)	83 (106–77)			36 (0)						36 (0)
**Central Mediterranean**										
Sardinia (SA)	135 (123–147)			8 (0)						8 (0)
Adriatic Sea (AS)	122 (110–133)			47						47 (0)

### Otolith stable isotope analysis

Otolith handling followed the protocols described in Rooker et al.^54^. Briefly, following extraction, sagittal otoliths were cleaned of excess tissue with nitric acid (1%) and deionized water. One sagittal otolith from each individual was embedded in Struers epoxy resin (EpoFix) and sectioned using a low speed ISOMET saw to obtain 1.5 mm transverse sections that included the core. Following attachment to a sample plate, the portion of the otolith core corresponding to approximately the yearling periods of ABFT was milled from the otolith section using a New Wave Research MicroMill system. A two-vector drill path based upon otolith measurements of several yearling ABFT was created and used as the standard template to isolate core material following Rooker et al.^35^. The pre-programmed drill path was made using a 500 µm diameter drill bit and 15 passes each at a depth of 50 µm was used to obtain core material from the otolith. Powdered core material was transferred to silver capsules and later analyzed for δ^13^C and δ^18^O on an automated carbonate preparation device (KIEL-III Thermo Fisher Scientific, Inc., Waltham, Mass.) coupled to a gas-ratio mass spectrometer (Finnigan MAT 252 Thermo Fisher Scientific, Inc.) at the University of Arizona. Stable isotopes of carbon and oxygen (δ^13^C and δ^18^O) are reported relative to the PeeDee belemnite (PDB) scale after comparison to an in-house laboratory standard calibrated to PDB.

### Genetic analysis

Samples were prepared for genetic analysis according to the protocol described by Rodríguez-Ezpeleta et al.^34^. Briefly, a piece of muscle tissue ~ 1 cm^3^ in size was excised from each fish and immediately stored in RNA-later or 96% molecular grade ethanol at − 20 °C. Genomic DNA was extracted from ~ 20 mg of tissue using the Wizard Genomic DNA Purification kit (Promega, WI, USA) following manufacturer’s instructions for “Isolating Genomic DNA from Tissue Culture Cells and Animal Tissue”. Extracted DNA was suspended in Milli-Q water and concentration was determined with the Quant-iT dsDNA HS assay kit using a Qubit 2.0 Fluorometer (Life Technologies). DNA integrity was assessed by electrophoresis, migrating about 100 ng of GelRed-stained DNA on an agarose 1.0% gel. Each individual was genotyped using the 96 SNP panel developed by Rodríguez-Ezpeleta et al.^34^ for ABFT using 96.96 Dynamic Array IFCs, and the resulting data was analyzed with the Fluidigm Genotyping Analysis Software.

### Data analysis

#### Characterising the spawning populations

The adult baseline samples were used to characterise the GoM and Med spawning areas using three approaches: (1) classification based on genetic data; (2) classification using isotope data; (3) classification using both isotope and genetic data together (integrated model). The otolith core stable isotope signatures of the adult baselines were compared to those of the yearling baseline (12–18 months old) used by Rooker et al.^29^ which comprised 115 individuals from western nursery areas and 150 individuals from eastern nurseries.

Classification was conducted by random forest analysis using the R package randomForest^55^ in R 3.5.2^56^. Random forest is an ensemble machine learning approach which has been shown to perform well compared to other classification methods when applied to otolith chemistry data and is not constrained by assumptions of normality and within-group homogeneity^57^. The method can deal with both continuous and categorical predictors and so is well suited to the integration of otolith chemistry and genetic data. In random forest analysis, decision trees are built by repeatedly subsampling the data through bootstrapping and using a randomly selected set of predictor variables. Each tree is used to assign the “out-of-bag” observations (those not included in the bootstrap sample) to a class based on the predictor variables. Observations are assigned to the class which obtains the majority of “votes” across all of the trees. Misclassification rates for the out-of-bag observations are used to determine the relative importance of each predictor variable for distinguishing between groups^58,59^. Variables were selected for inclusion in the models based on out-of-bag error estimates and variable importance, as indicated by the mean decrease in the Gini coefficient (higher values indicate greater variable importance). Variables which had a negligible influence on the out-of-bag error and which had a low mean decrease in the Gini coefficient were excluded from the models. The variable selection process was automated using the VSURF package in R^60^. Interactions between predictor variables were explored using the iml package in R^61^.

#### Assigning individuals in the mixed sample to stock of origin

Individuals in the mixed sample for which both genetic and isotope data were available were assigned to their population of origin by random forest using the integrated classification model (isotopes and genetics) and the isotope only model. Population assignments were compared with the assignments previously derived by Rodríguez-Ezpeleta et al.^34^ for the same individuals and with assignment based on quadratic discriminant function analysis of the yearling baseline^29^.

#### Simulation of population mixtures

The population assignment step provided estimates of the population mixture in each area which varied depending on the method used. A series of simulations were run in order to compare the observed distribution of otolith δ^18^O values in the mixed sample to the distribution that would be expected if the mixture was selected at random from the same populations as the adult baselines. Three density distributions were generated using the distr package in R^62^ by drawing 1,000,000 random samples from a two component mixture distribution with means and standard deviations equal to the Med and GoM populations in the adult baseline and with mixing coefficients equal to those estimated using (1) the genetic assignment method from Rodríguez-Ezpeleta et al.^34^; (2) assignments based on the yearling baseline of Rooker et al.^29^; (3) random forest predicted assignments using the adult baseline from this study and (4) random forest predicted assignments using the integrated model from this study.

#### Broad scale estimates of δ^18^O in seawater and otoliths

The isotopic composition of oxygen in otoliths (δ^18^O_oto_) is linearly related to the isotopic composition (δ^18^O_w_) and temperature (T) of the seawater in which the fish resides through the fractionation equation:

$$\delta^{18} {\text{O}}_{{{\text{oto}}}} - \delta^{18} {\text{O}}_{{\text{w}}} = \gamma {\text{T}} + \beta .$$

By coupling an empirically derived fractionation equation with estimates of δ^18^O_w_ and sea surface temperature (SST) it is possible to use otolith oxygen isotope ratios as geolocators, although the successful application of this approach is somewhat limited by uncertainties surrounding small scale variation in δ^18^O_w_ and species specific variation in the γ and β parameters^63^. The measured δ^18^O values in the otolith core of adult ABFT reflect the average temperature and δ^18^O_w_ conditions experienced by the fish during the first 12 months of life with a bias towards the period in which growth is fastest (July–October^64^). The isoscape approach was used to produce a map of mean predicted δ^18^O_oto_ in July–October by combining spatially resolved estimates of δ^18^O_w_ and SST via a fractionation equation. A 1° × 1° grid of δ^18^O_w_ was obtained from the dataset published by LeGrand and Schmidt^65^. Long term monthly mean estimates of SST for the period 1981–2010, calculated at the same spatial resolution, were obtained from the COBE SST data provided by the NOAA/OAR/ESRL PSD, Boulder, Colorado, USA, from their Web site at https://www.esrl.noaa.gov/psd/^66^. The fractionation equation for Pacific bluefin tuna (*Thunnus orientalis*)^67^ was used to estimate δ^18^O_oto_ at each point on the 1° × 1° grid. These estimates of δ^18^O_oto_ were then mapped using interpolation. From this isoscape, the mean and range of expected δ^18^O_oto_ values were calculated for ten regions in which larval and yearling ABFT are known to occur^38–40,68^ and for the Central Atlantic which is the main route of trans-Atlantic migration^32^. The aim was not to determine the exact location in which each individual resided during their first year, but to capture broad scale variation between regions against which hypotheses relating to early movement could be evaluated.

## Results

### Comparison of adult and yearling baselines

**Figure 2:**
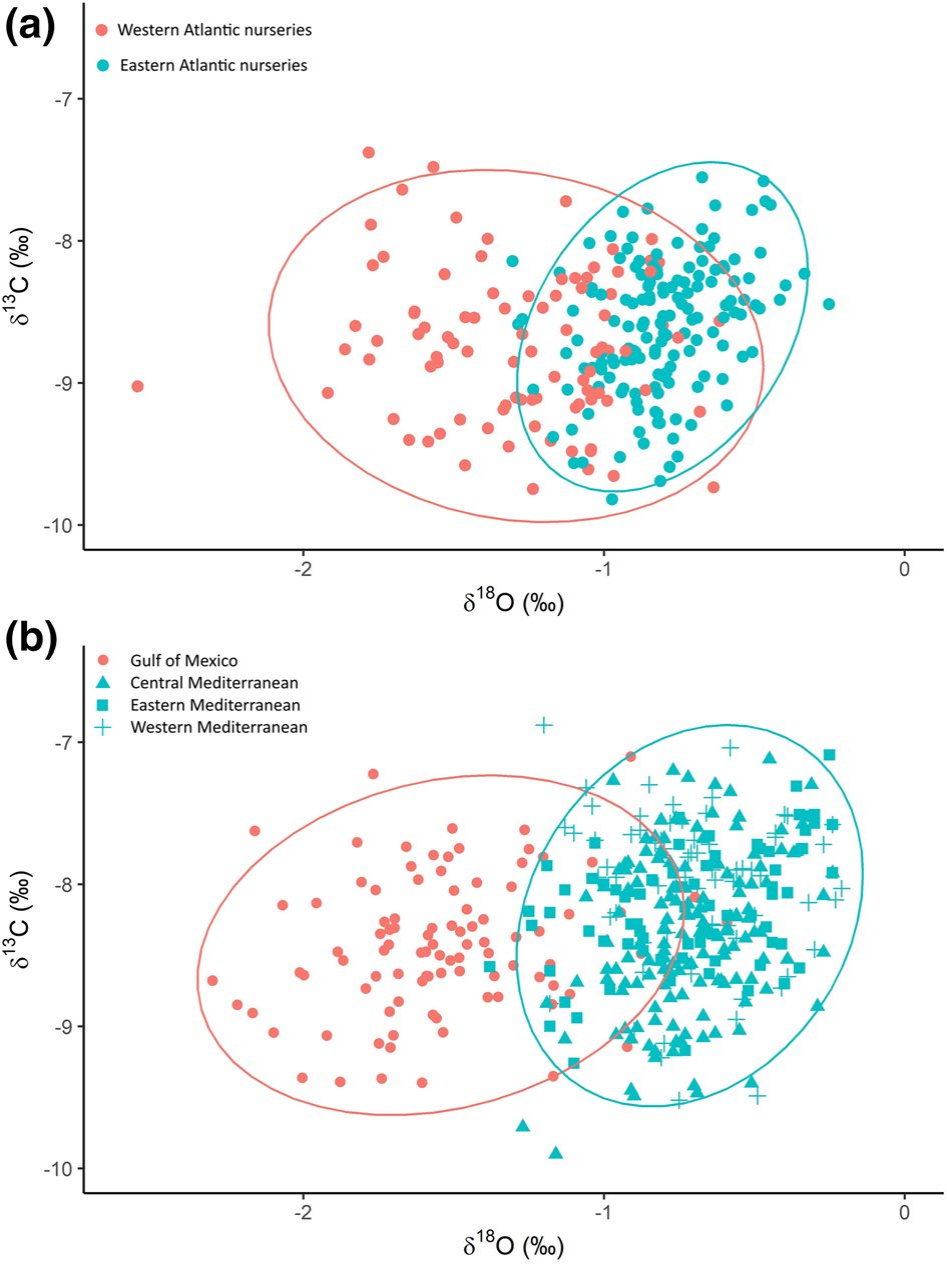
Otolith core values of δ^13^C and δ^18^O for the yearling baseline samples used by Rooker et al.^29^ (a) and for the adult baseline samples used in this analysis (b). 95% confidence ellipses are shown for each population.

ABFT collected during the spawning season from the GoM and the Med had distinct otolith core isotope signatures (Fig. 2a) which showed less overlap than those of yearling ABFT collected from nurseries in the eastern and western Atlantic (data from Rooker et al.^29^; Fig. 2b). Stable isotope signatures were similar in adults collected from the western, central and eastern Mediterranean Sea.

When an assignment threshold of 50% was used, each individual in the baseline samples was assigned to the population with the majority of “votes” from the trees in the random forest. When the assignment threshold was set to 80%, individuals were assigned to a population if over 80% of the trees predicted that it belonged to that population and were unassigned if the votes for both populations were < 80%.

With a 50% assignment threshold, fish from the adult chemistry baseline were classified to their population of origin based on δ^18^O and δ^13^C otolith core values with a mean accuracy of 93.8%. When an assignment threshold of 80% was used, 84.3% were correctly classified, 2.6% were incorrectly classified and 13% could not be assigned to either population (Table 3a). Rates of classification accuracy were lower for the yearling baseline; 81.7% were assigned to the correct population with a probability threshold of 50%. When the probability threshold was set to 80%, 65.3% were correctly assigned, 4.2% were incorrectly assigned and 30.6% were unassigned (Table 3b). This confirmed that otolith core stable isotope signatures of mature adults at the two main spawning areas are more discrete than those of yearling fish collected from nursery areas in the western and eastern Atlantic. In both models, the most important variable for distinguishing between the groups was δ^18^O (Table 4). For the yearling baseline, δ^13^C improved the overall classification accuracy of the model; for the adult baseline δ^13^C improved classification accuracy for the GoM population but not the Med population. The random forest approach incorporates interactions between predictors so that the effect of one predictor depends on the value of the other predictor^69^. The H-statistic which varies from 0 to 1, measures interactions between predictors; a value of 1 indicates that all of the variability in prediction due to that predictor is explained by interactions with other predictors. Interactions between δ^18^O and δ^13^C had a stronger influence on predictions in the yearling baseline model compared to the adult baseline model (Table 4).

**Table 3:** Confusion matrix from the random forest analysis using δ^13^C and δ^18^O isotope measurements a) from the adult baseline and b) from the yearling baseline samples^29^.

(a) Isotopes—adult baseline				
True origin	Estimated origin			
	GoM	Med	UA	%correct
GoM	85 (77)	9 (7)	0 (10)	90.4 (81.9)
Med	15 (3)	275 (247)	0 (40)	94.8 (85.1)
Total	100 (80)	284 (254)	0 (50)	93.8 (84.3)

(b) Isotopes—yearling baseline				
True origin	Estimated origin			
	West	East	UA	%correct
West	94 (74)	21 (5)	(36)	81.7 (64.3)
East	25 (6)	125 (99)	(45)	83.3 (66)
Total	119 (80)	146 (104)	(81)	82.6 (65.3)

Figures relate to the numbers of individuals from the baseline samples assigned to the Gulf of Mexico (GoM) and Mediterranean (Med) spawning populations or unassigned (UA) using bootstrap sub-sampling and probability thresholds of 50% (outside parenthesis) and 80% (inside parenthesis).

**Table 4:** Summary of variable importance measures from the random forest models.

Model	OBE %	Variable	Variable importance				Variable interactions
			Mean decrease in Gini coefficient	Mean decrease in accuracy			H-statistic
				Med	GoM	Mean	
Adult chemistry baseline (full chemistry dataset)	6.25	δ^18^O	78.4	104.2	186.1	145.43	0.04
		δ^13^C	15.6	-3.5	4.5	-1.14	0.17
Yearling chemistry baseline (dataset from Rooker et al.^29^)	17.4	δ^18^O	82.6	127.4	157.2	172.3	0.47
		δ^13^C	32.1	13.7	29.8	27.3	0.51
Adult chemistry baseline (combined dataset)	4.1	δ^18^O	32.1	61.5	90.1	81.7	0.03
		δ^13^C	11.9	0.66	9.0	6.1	0.31
Genetics (combined dataset)	9.0	RAD 26	4.3	27.6	27.5	31.5	0.49
		RAD 213	11.0	59.4	52.6	60.3	0.48
		RAD 35	7.6	54.8	43.7	54.1	0.42
Integrated model (combined dataset)	3.5	δ^18^O	26.2	58.8	73.3	77.6	0.36
		RAD 213	7.5	18.6	32.5	33.2	0.23
		RAD 26	1.8	10.6	5.6	10.5	0.19
		RAD 35	4.4	8.5	17.7	18.2	0.02
		RAD 2	1.9	4.3	5.4	6.6	0.11

### Classification of adult baseline samples: comparison of methods

Adults from the combined baseline (both genetic and chemistry data available) could be accurately discriminated based on δ^13^C and δ^18^O isotope values (overall classification success 95.9%) or three SNP genetic markers (overall classification success 91.0%) when an assignment threshold of 50% was used. However, the stable isotope approach was more powerful for classifying the GoM fish (91.1%) compared to the genetic approach (77.3%). When an 80% assignment threshold was applied, 83.4% were correctly classified by the isotope model, 2.8% were incorrectly classified and 13.8% were unassigned. The genetics model correctly assigned 69.7% of individuals, incorrectly assigned 4.8% and was unable to assign 25.5%. Rates of correct assignment were similar for the Med population but the genetics model correctly assigned only 36.3% of the GoM fish (compared to 81.8% for the isotopes model).

The highest rates of classification success for both populations were achieved using a combination of otolith chemistry and genetics (δ^18^O, RAD213, RAD26, RAD35 and RAD2) (Table 5). Across both populations, the combined model correctly classified 96.6% of individuals with a 50% threshold. When an 80% threshold was applied, 89% were correctly classified, 1.4% were incorrectly classified and 9.7% were unassigned. Improvements relative to the chemistry only model were marginal when a threshold of 50% was used but were more marked when the 80% threshold was applied, particularly for the Med population (91.1% and 84.2% correct for the combined and isotope models, respectively). The accuracy of the combined model far exceeded that of the genetics only model for the GoM population; with an 80% threshold, 84.1% were correctly assigned by the combined model compared to 36.3% by the genetics only model.

**Table 5:** Confusion matrix from the random forest analysis, using (a) δ^13^C and δ^18^O isotope measurements (b) three SNP genetic markers (RAD213, RAD26 and RAD35) and c) a combination of otolith chemistry and genetics (δ^18^O, RAD213, RAD26, RAD35 and RAD2) to discriminate between adult Atlantic bluefin tuna from spawning populations in the Gulf of Mexico and the Mediterranean.

True origin	Estimated origin			
	GoM	Med	UA	%correct
**(a) Isotopes—adult baseline**				
GoM	41 (36)	3 (3)	0 (5)	93.2 (81.8)
Med	3 (1)	98 (85)	0 (15)	97.0 (84.2)
Total	44 (37)	101 (88)	0 (20)	95.9 (83.4)
**(b) Genetics—adult baseline**				
GoM	34 (16)	10 (7)	0 (21)	77.2 (36.3)
Med	3 (0)	98 (85)	0 (16)	97.0 (84.2)
Total	37 (16)	108 (92)	0 (37)	91.0 (69.7)
**(c) Isotopes and genetics—adult baseline**				
GoM	42 (37)	2 (1)	(6)	95.5 (84.1)
Med	3 (1)	98 (92)	(8)	97.0 (91.1)
Total	45 (37)	100 (93)	(14)	96.6 (89.0)

Figures relate to the numbers of individuals from the baseline samples assigned to the Gulf of Mexico (GoM) and Mediterranean (Med) spawning populations or unassigned (UA) using bootstrap sub-sampling and probability thresholds of 50% (outside parenthesis) and 80% (inside parenthesis). One individual from the GoM and four individuals from the Med were excluded from the analysis due to missing genetic data.

Variable importance measures (Table 4) showed that δ^18^O was the most important discriminator for both populations across all models. δ^13^C improved the accuracy of predictions in the isotopes only model but was excluded from the combined model through variable selection. In the combined model RAD213 was the second most important discriminator, followed by RAD26, RAD35 and then RAD2. Interactions between predictors explained more of the variance in the genetics and integrated models than in the chemistry only model (Table 4). Notably, in the integrated model, 36% of the variance in predictions due to δ^18^O was explained by interactions with the genetic variables. In other words, the extent to which a change in δ^18^O influences the probabilities of an individual belonging to each spawning population depends on the genotype of that individual.

### Population assignment of mixed sample

A total of 750 fish of unknown stock origin (the mixed sample) had been analysed using both the genetic and stable isotope methods. Of these, 707 had usable data for both stable isotope markers and the four SNP markers that were used in the combined model. The proportions of these fish that were assigned to each population using the stable isotope model and integrated model from this study as well as the genetic method (96 SNPs) of Rodríguez-Ezpeleta et al.^34^ and the stable isotope method (yearling baseline) of Rooker et al.^29^ are shown in Table 6.

**Table 6:** Proportions of Atlantic bluefin tuna  in the mixed samples assigned to each spawning population using each method of assignment.

Area	Years	Isotopes yearling baseline (Rooker et al.^29^)			Genetics (Rodríguez Ezpeleta et al.^34^)			Isotopes adult baseline			Integrated model (isotopes and genetics)			N
		Unassigned	WAtl	Med	Unassigned	WAtl	Med	Unassigned	WAtl	Med	Unassigned	WAtl	Med	
BB	2011, 2012	0.52	0.03	0.44	0.14	0.08	0.78	0.20	0.03	0.77	0.14	0.03	0.82	90
CA (E)	2011, 2012, 2013, 2014, 2015	0.37	0.16	0.47	0.09	0.03	0.88	0.32	0.08	0.59	0.26	0.08	0.65	159
CA (W)	2011, 2012, 2013, 2014, 2015	0.35	0.35	0.30	0.15	0.23	0.62	0.33	0.23	0.44	0.23	0.26	0.51	124
CI	2013, 2016	0.43	0.15	0.42	0.05	0.05	0.90	0.28	0.13	0.58	0.22	0.12	0.67	60
MO	2012, 2013, 2014, 2015, 2016	0.35	0.13	0.52	0.13	0.03	0.84	0.24	0.11	0.65	0.23	0.07	0.70	234
PO	2011, 2012	0.23	0.15	0.63	0.20	0.00	0.80	0.20	0.05	0.75	0.23	0.03	0.75	40

There were marked discrepancies in the proportions of the mixed sample that were assigned to the eastern and western stocks using the two methods that are currently used in stock assessment and MSE; 0–11% of the mixed samples were assigned to the western population using the genetic baseline developed by Rodríguez-Ezpeleta et al.^34^, while 3–24% were estimated to be of western origin using the yearling chemistry baseline from Rooker et al.^29^. In addition, the number of individuals that could not be assigned to either population with a probability of > 0.8 was higher when using the yearling baseline (23–52%) compared to genetics (5–20%). The use of the adult chemistry baseline for population assignment reduced the proportion of unassigned fish (20–33%) compared to the yearling baseline and the proportions assigned to the western population (3–15%) were closer to (but still higher than) the proportions assigned using genetics. Using the integrated model, rates of assignment to the western population were similar to those obtained using the isotope model for some areas (Bay of Biscay, Canary Islands and Central Atlantic) and were intermediate between the genetic and isotope estimates for others (Morocco and Portugal). For most areas (with the exception of Portugal), fewer fish were unassigned by the combined model (14–25%) compared to the isotopes only model. The proportion of unassigned fish was lower for the genetics model than for the combined model in all areas except the Bay of Biscay.

**Figure 3:**
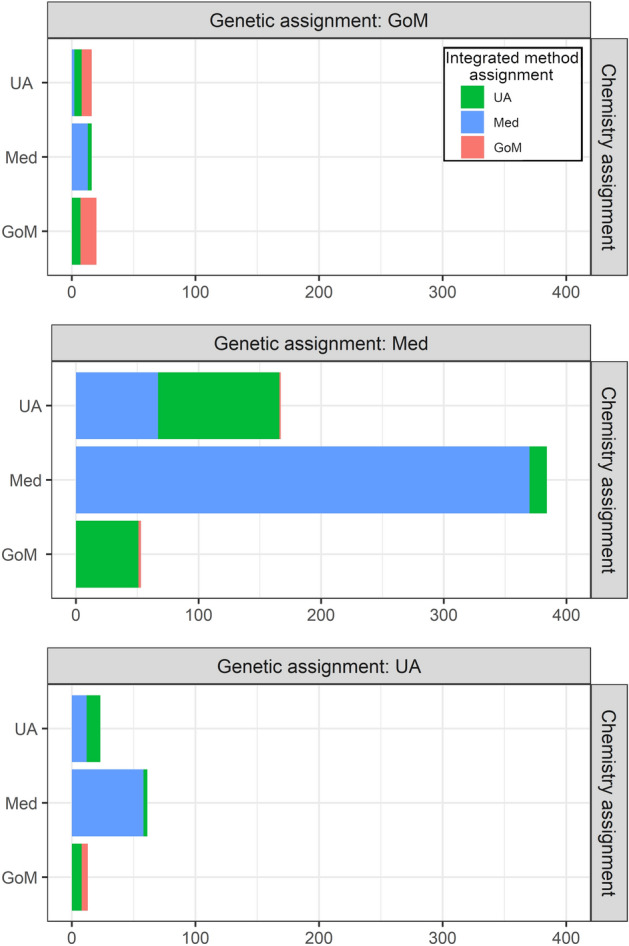
Three-way comparison showing the numbers of individuals in the mixed sample that were assigned the Mediterranean (Med) and Gulf of Mexico (GoM) spawning populations or were unassigned (UA) using each of the three approaches (genetics, chemistry and the integrated method).

A three way comparison of the genetics only, chemistry only and integrated model population assignments for the 707 individuals in the mixed sample is shown in Fig. 3.

The proportion of unassigned fish (probability of assignment < 80%) was 27% for chemistry only and 13% for genetics only. Most of the fish that were unassigned using one method could be assigned using the other; only 20 fish (2.8%) could not be assigned using either genetics or chemistry. Of these fish, 8 (1.1%) could not be assigned to either population by the integrated method.

For individuals that were assigned to the same population using both genetics and chemistry (17 to GoM and 360 to Med) the integrated method also assigned them to that population, with the exception of 15 fish which were unassigned (1 of the GoM and 14 of the Med assignments). In this case, the lower rate of assignment for the integrated method most likely reflects the smaller sample size of the combined baseline, particularly for the GoM population.

**Figure 4:**
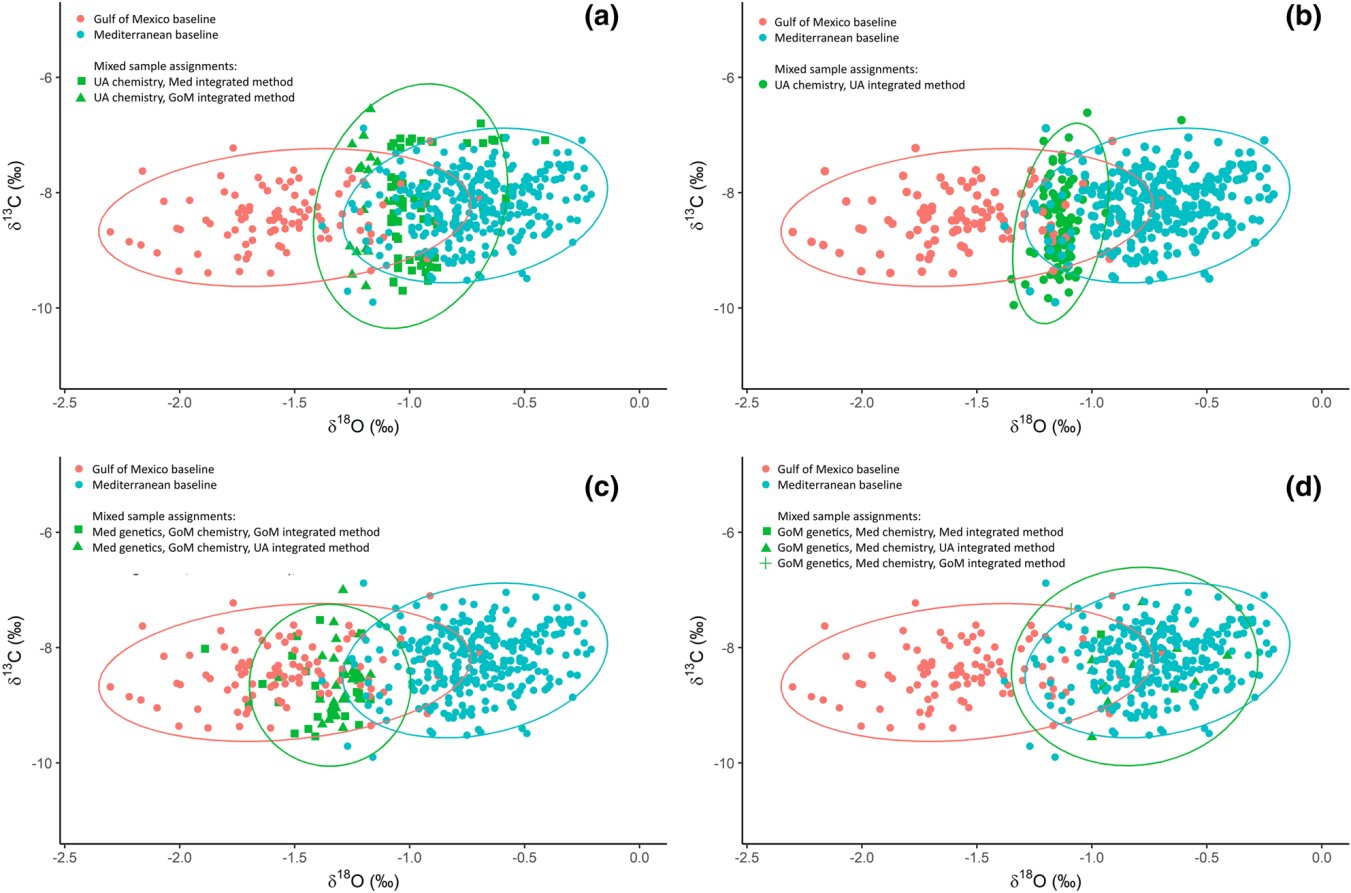
Otolith core values of δ^13^C and δ^18^O for individuals of disputed origin from the mixed sample, grouped according to how they were assigned using each of the three methods and overlaid on the otolith core stable isotope signatures of the adult baselines.

A total of 192 fish had stable isotope signatures that lay in the area of overlap between the two baseline populations and were unassigned using the chemistry only model; 94 of these were positively assigned using the integrated model; 74 to the Med and 20 to the GoM (Fig. 4a). The mean δ^18^O isotope values for these fish was − 1.0‰; intermediate between the mean values for the GoM (− 1.5‰) and the Med (− 0.7‰). The remaining 98 fish which could not be assigned using either the stable isotope or integrated method had lower δ^18^O isotope values (mean = − 1.1‰; Fig. 4b).

The highest rate of discrepancy in the single method assignments was for the fish that were assigned to the GoM using stable isotopes: 65% of these 81 fish were assigned to the Med using genetics, 21% were assigned to GoM using genetics and 14% were unassigned. Just over half (53%; 28 fish) of the fish which were assigned to the GoM using stable isotopes and to the Med using genetics could not be assigned using the integrated method. These individuals had stable isotope signatures that overlapped with the GoM baseline (Fig. 4c) but their δ^18^O isotope values were higher than the mean for that population (− 1.3‰). The remainder of the fish that were assigned to the GoM using stable isotopes and to the Med by genetics were assigned to the GoM by the integrated method (47%, 25 fish). The mean δ^18^O isotope values for these fish overlapped with those that were unassigned by the integrated method but their mean was closer to the mean for the GoM population (− 1.4‰).

The genetics method assigned 48 fish to the GoM population; 17 of these had otolith δ^18^O values that were typical of the GoM baseline (mean δ^18^O = − 1.5‰) and were also assigned to the GOM using stable isotopes. However, 16 had otolith δ^18^O values that were more typical of the Med baseline; these were assigned to the Med using chemistry (mean δ^18^O = − 0.8‰); and were either assigned to the Med (four individuals) or unassigned (11 individuals) using the integrated method (Fig. 4d). Only one individual that was assigned to the GoM by genetics and to the Med using stable isotopes was assigned to the GoM using the integrated method.

Overall, the integrated method increased the rate of positive assignments relative to the chemistry method for fish with otolith δ^18^O values in the area of overlap between the two baselines and helped to resolve discrepancies between the stable isotope and genetics methods. Fish with Med-like genetics and GoM-like stable isotope signatures were assigned to the GoM population when their otolith δ^18^O values were close to the mean for that population. Fish with GoM-like genetics and Med-like chemistry had otolith δ^18^O values that were very typical of the Mediterranean baseline and most could not be assigned to either population by the integrated method.

### Simulation of population mixtures

**Figure 5:**
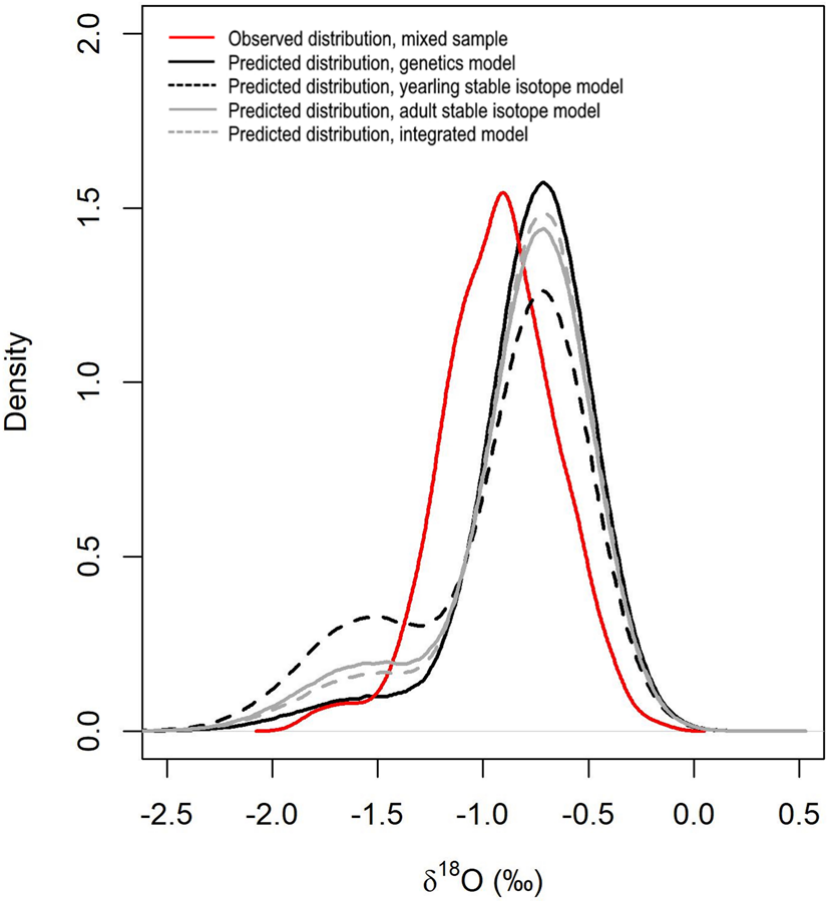
Density distributions of otolith core δ^18^O values in the mixed sample (N = 707) overlaid on predicted density distributions under the scenario that the mixed sample is randomly drawn from the adult baseline in the proportions indicated by the genetic method assignments; chemistry yearling baseline assignments; chemistry adult baseline assignments and integrated method assignments. Predictions were generated by drawing 1,000,000 random samples from a two component mixture distribution with means and standard deviations equal to the Mediterranean and Gulf of Mexico populations in the adult baseline and with mixing coefficients equal to those indicated by each assignment method.

The density distribution of δ^18^O values in the simulated population mixtures did not align well with the observed δ^18^O values in the mixed sample for any of the estimated mixing rates (Fig. 5). The distributions suggest that there is a disproportionately higher number of fish with δ^18^O values of between − 1.2 and − 1.4‰ than would be predicted if these samples were randomly drawn from the same populations as the adult baseline (mode of the observed distribution sits to the left of the predicted distribution). The proportion of fish in the mixed sample with δ^18^O values between − 1.5 and − 2.0‰ (typical of the GoM baseline) was similar to what would be expected given the predictions from the genetics model and lower than would be expected given the predictions from the integrated model and the two chemistry models, particularly the yearling baseline model.

### Distribution of δ^18^O values in the full mixed dataset

**Figure 6:**
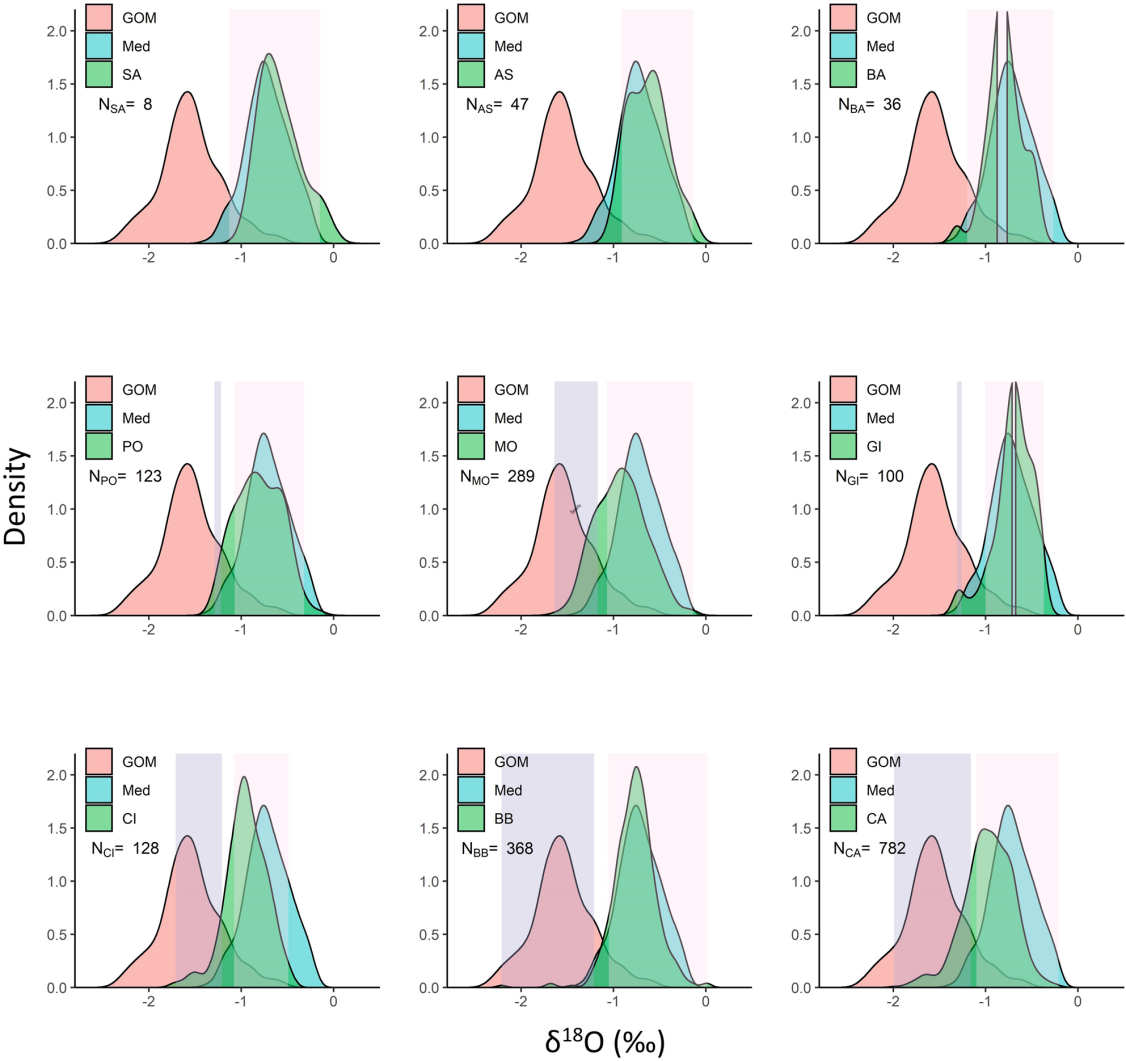
Density distributions of δ^18^O values in the otolith cores of Atlantic bluefin tuna  (ABFT) of unknown spawning origin (green) collected from the Mediterranean (SA, AS, BA) and from the eastern (PO, MO, CI, GI, BB) and central (CA) Atlantic overlaid on the density distribution of δ^18^O values in the otolith cores of adult ABFT from the main spawning areas in the Gulf of Mexico (pink) and the Mediterranean (blue). The shaded areas indicate the range of δ^18^O values of the fish that could be assigned to either population with a probability > 0.8 using the adult baseline stable isotopes model (grey shading—GoM; pink shading—Med).

In the full mixed dataset (N = 2031) the distribution of δ^18^O values in samples of juveniles and non-spawning adults collected in the west Mediterranean (BA), central Mediterranean (SA, AS), Bay of Biscay (BB) and Gibraltar (GI) were closely aligned with the distribution of δ^18^O values in the adult baseline samples. For samples from the central Atlantic (CA), Canary Islands (CI), Morocco (MO) and to a lesser extent Portugal (PO), the mode of the distribution of δ^18^O values lay between the modes of Med and GoM baselines; the proportion of individuals with δ^18^O of between − 1.2 and − 1.4‰ was higher than the proportions observed in either of the baseline populations (Fig. 6).

### Predicted mean values of δ^18^O in otoliths

**Figure 7:**
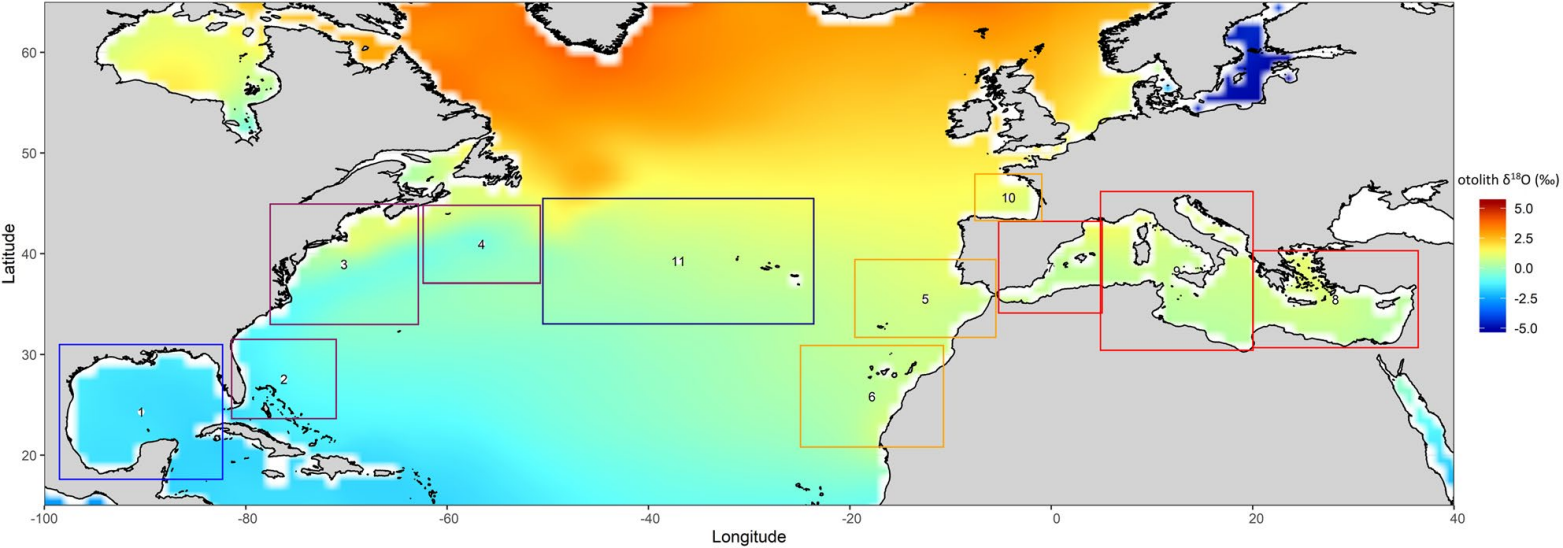
Isoscape of predicted δ^18^O in otoliths based on mean annual estimates of δ18O at the surface of the water^65^, mean SST during the period of rapid growth (July–October)^66^ and the fractionation equation for Pacific bluefin tuna^67^. Map was created in R using the ggplot2 package version 3.2.1 URL: https://ggplot2.tidyverse.org^79^.

The δ^18^O isoscape displayed broad east–west and north–south gradients in the predicted levels of δ^18^O in the otolith (Fig. 7) which reflect the combined influence of spatial variation in δ^18^O of seawater and gradients in SST. As expected, the isoscape indicated that fish residing in the Mediterranean Sea would have otoliths that were enriched in ^18^O relative to those residing in the Gulf of Mexico. Overall, the predicted values were higher than what is observed in the otolith cores of ABFT (Table 7). In western Atlantic areas outside of the Gulf of Mexico, where spawning activity may also occur (Bahamas, and Slope Sea)^38–40^, predicted δ^18^O values in the otolith were intermediate between those of the Mediterranean Sea and Gulf of Mexico. For areas in the eastern Atlantic where yearling ABFT  reportedly occur (Canary Islands, Gibraltar, Bay of Biscay), predicted δ^18^O in otoliths were elevated relative to both the Gulf of Mexico and the Mediterranean Sea. In the Central Atlantic predicted otolith δ^18^O values were similar to those for the Mediterranean Sea.

**Table 7:** Predicted mean and range of δ^18^O in otoliths based on mean annual estimates of δ^18^O at the surface of the water^65^, mean SST during the period of rapid growth (July–October) and the fractionation equation for Pacific bluefin tuna^67^.

Area number	Location	Predicted δ^18^O in otoliths (‰)		
		Mean	Minimum	Maximum
1	Gulf of Mexico	− 1.84	− 1.93	− 1.71
2	Bahamas	− 1.45	− 1.79	− 1.10
3	Slope Sea west	− 0.83	− 1.45	− 0.14
4	Slope Sea east	− 0.88	− 1.45	0.25
5	Gibraltar	0.32	0.10	0.74
6	Canaries	0.03	− 0.41	0.55
7	Western Mediterranean	0.05	− 0.20	0.80
8	Eastern Mediterranean	0.03	− 0.40	0.59
9	Central Mediterranean	− 0.03	− 0.45	0.75
10	Bay of Biscay	0.75	0.37	1.11
11	Central Atlantic	− 0.08	− 0.76	0.78

## Discussion

In this study, genetic (SNPs) and environmental (otolith core stable isotopes) markers of stock origin were combined to resolve uncertainty surrounding movement and population mixing of ABFT. Combining stable isotopes with genetics in an integrated population discrimination model improved the accuracy of assignments to the adult baseline populations relative to the single marker methods, particularly with respect to genetic assignment of the GoM population. ABFT from the mixing areas in the Central and Eastern Atlantic which could not be assigned to their population of origin using one method could usually be assigned using the other, highlighting the value of amalgamating information from genetics and stable isotopes. For some individuals, the integrated model helped to resolve discrepancies between the genetic and stable isotope methods while other individuals remained unassigned. The combined approach is therefore useful for identifying individuals whose stable isotope signatures and genetic profiles, when taken together, are not characteristic of either the western or eastern spawning population. The occurrence of these individuals in the mixing areas may indicate a potential weakness in the two stock population model for ABFT.

An important finding of this study was that the otolith core stable isotope signatures of the adult baselines were more distinct than those of the yearling baselines reported by Rooker et al.^29^. This is unlikely to reflect methodological differences as the same protocols were adopted in the two studies and the analysis was conducted using the same machine. More sample preparation is required to isolate the core (corresponding to the first 12 months of life) of an adult otolith compared to an otolith from a yearling ABFT and the risk of including material deposited after the first year of life in the core section is higher. Given the extensive trans-Atlantic migrations undertaken by ABFT from both stocks after the yearling phase^31,32^, this potential source of error would more than likely make otolith core signatures of adults more similar rather than more distinct than those of yearlings.

The temporal coverage of the baseline samples should also be considered; otolith core stable isotope signatures show annual variability due to local climatic effects on seawater δ^18^O and δ^13^C. Seawater δ^13^C values have declined in recent decades due to the increase in atmospheric concentrations of fossil fuel derived CO_2_, which is depleted in δ^13^C (a phenomenon known as the Suess effect^70^). This could introduce some limited temporal variability to δ^13^C levels in the otolith, which reflects δ^13^C in seawater as well as dietary carbon, with the relative contribution of each modulated by temperature^71^. However, the implications of such variability for this study are minimal as the discrimination of eastern and western ABFT is based almost entirely on δ^18^O. Variability in seawater δ^18^O is minor relative to the variability between the eastern and western nursery areas and so otolith core stable isotope signatures are thought to provide a temporally stable population marker^29,35^. The yearling baseline samples used by Rooker et al.^29^ were 12–18 months old and were collected from western nurseries between 1998 and 2007 and from eastern nurseries between 2000 and 2011. Otolith-based age estimates for the GoM adults used in this study indicate that the fish were between seven and 30 years old and had hatched between 1979 and 2005. The Med adults were between four and 22 years old according to length-based age estimates from the Von Bertalanffy growth model^72^ and so had hatched between 1988 and 2008. As most of the year-classes included in the yearling baseline are also represented in the adult baseline, the greater overlap in the otolith core stable isotopes of the yearling baseline cannot be explained by temporal variability.

The difference between the yearling and adult baselines may reflect early exchange between nursery areas or the existence of a third spawning component. Trans-Atlantic migrations of ABFT are age dependant and are only rarely recorded in juveniles; a review of conventional tagging studies shows that for fish tagged at between one and four years of age and at liberty for less than 1 year, less than 1% of recaptures had crossed the Atlantic^68,73^. Given that the eastern Atlantic stock is about one order of magnitude larger than the western Atlantic stock^28^, low rates of trans-Atlantic movement during the first 18 months of life are unlikely to be detectable in the eastern Atlantic but could impact on stock composition at western nursery grounds. Isoscape predictions (Fig. 7, Table 7) suggest that if a fish from the Mediterranean spawning area crossed the Atlantic, δ^18^O in the otolith would remain elevated relative to Western Atlantic residents for much of its migration. It is therefore conceivable that some yearlings at western nurseries with intermediate otolith core oxygen stable isotope values (δ^18^O ~ − 1) are of Mediterranean origin. Similarly, the isoscape predictions indicate that δ^18^O in the otoliths of ABFT originating from the potential spawning area in the Slope Sea would be intermediate between those originating from the Mediterranean and the Gulf of Mexico. More in-depth knowledge of ABFT distribution during the larval and juvenile phases is needed to fully resolve the larval origin of fish at western and eastern nurseries. Electronic tagging studies show strong fidelity of individual ABFT to spawning areas in the Gulf of Mexico and Mediterranean Sea^32^ while genetic evidence supports natal homing^34^. Although 100% fidelity to natal areas cannot be assumed, spawning group membership of mature adults collected from the two main spawning areas during the spawning season can be more definitively determined from catch location than that of yearlings in nursery areas. It is recommended that future population assignment studies use the adult baselines to characterise the Mediterranean and Gulf of Mexico populations when applying the stable isotope approach.

Genetic SNP markers have been proposed as a cost-effective and non-invasive population traceability tool with accuracy rates that are comparable to the otolith core stable isotope method^29^, but without the need to sacrifice the fish^34^. The results of this study highlight the advantages of using stable isotopes in concert with genetic data to provide a more accurate and complete picture of stock origin. The discriminatory power of otolith core stable isotope signatures (from the adult baselines) is similar to that achieved with the full panel of SNP markers. Using an 80% assignment threshold, Rodríguez-Ezpeleta^34^ report a correct assignment rate of 81% for the Gulf of Mexico and 83% for the Mediterranean populations, with 10% and 2% incorrectly assigned and 9% and 15% unassigned respectively. Using the stable isotope adult baseline with an 80% assignment threshold, 82% and 85% were correctly assigned, 7% and 1% were incorrectly assigned and 11% and 14% were unassigned (Gulf of Mexico and Mediterranean Sea, respectively). The integrated method, which combines four genetic markers with δ^18^O, classifies the Mediterranean population more accurately and with fewer unassigned individuals than any other method; with an 80% assignment threshold, 91% of the Mediterranean individuals were correctly assigned, 1% were incorrectly assigned and 8% were unassigned. For the Gulf of Mexico population the rate of correct assignment is marginally higher (84%) than that reported by Rodríguez-Ezpeleta^34^ while fewer individuals are incorrectly assigned (2%) and more are unassigned (14%). Overall, the integrated method can distinguish between the western and eastern stocks of ABFT more accurately and with a lower risk of incorrect assignment than either stable isotopes or genetics alone. Its superior performance justifies the costs associated with collecting stable isotope data in addition to genetic data. One potential limitation of the results presented here is the restricted size of the Gulf of Mexico combined baseline that was used to develop the integrated model (N = 44). The expansion and refinement of this baseline should be prioritised through co-ordinated sampling of adults in the Gulf of Mexico spawning area during the spawning season.

Our results demonstrate that at the individual level, genetic and stable isotope markers can provide contradictory estimates of stock origin, particularly for catches from the Canary Islands and Morocco. This uncertainty is partly resolved by the integrated method due to properties of the random forest model which allow for the incorporation of interactions between predictors. Some individuals of disputed origin are assigned to one population using the integrated model because their stable isotope and genetic profile, when taken together, are more closely aligned to that baseline. For others their combined genetic and stable isotope profiles were not characteristic of either baseline population and they remained unassigned.

Individuals that were assigned to the Med population using genetics and to the GoM using stable isotopes and which could not be assigned to either using the integrated method had otolith core δ^18^O values between − 1.2 and − 1.4‰; which is at the higher end of the range for the GoM baseline and overlaps with the extremes of the Med baseline (4% of Med baseline). The proportion of individuals with this type of δ^18^O in the mixed samples was higher than predicted based on the distribution of δ^18^O in the baseline samples, particularly in samples from the Canary Islands, Morocco, the Central Atlantic and (less so) Portugal. The results indicate that regardless of the mixing proportions, the fish occurring in these areas do not represent a mixture that is randomly drawn from the western and eastern stocks of ABFT. This could occur if the adult baseline samples were not fully representative of the fish that spawn in the Gulf of Mexico or the Mediterranean Sea in May and June. This is unlikely given the temporal and spatial coverage of the baseline samples. In addition, the stable isotope signatures of the Mediterranean baseline align closely with those of adult and juvenile fish (size range 52.5–293 cm) collected from many of the sites within the Mediterranean east Atlantic from 2009 to 2016.

ABFT with δ^18^O values of between − 1.2 and − 1.4‰ may include fish from a third component that spawns at another location or at a different time of year. This is consistent with the isoscape predictions which suggest intermediate levels of δ^18^O in the otoliths of ABFT originating from the Slope Sea and Bahamas where some evidence of spawning has been detected^38–40^. It has been proposed that ABFT spawning in this area are from the same population as those spawning in the Gulf of Mexico and that movement of fish between the two spawning grounds is size dependent^39^. However, Rodríguez-Ezpeleta et al.^34^ report genetic differentiation between larvae from the Gulf of Mexico and Slope Sea young of the year. Alternatively, the individuals with intermediate δ^18^O values may represent a migratory contingent of the Mediterranean population which diverges from the resident population during their first year of life and which is more likely to occupy mixing areas in the eastern and central Atlantic during adulthood. At spawning time, otolith chemistry signatures for this contingent could be diluted by mixing with the rest of the Mediterranean population. This hypothesis is consistent with evidence from electronic tagging studies which suggests that the Mediterranean population comprises a resident and a migratory component^53,74^ which both spawn in the western and central Mediterranean^75^ and possibly the eastern Mediterranean^76^. The isoscape predictions presented here suggest that movement of ABFT to the Bay of Biscay and North coast of Africa would increase otolith δ^18^O but movement from the Mediterranean to more southern areas of the East Atlantic or to the West Atlantic during the first year could decrease δ^18^O values in the otolith core. Currently, knowledge of early movements of ABFT is not sufficiently well developed to fully evaluate this hypothesis. More detailed investigation of δ^18^O across otolith transects, combined with a comprehensive genetic analysis could help to clarify the origin and movements of ABFT with intermediate δ^18^O values.

Population models for ABFT are highly sensitive to estimates of stock mixing. While the distributions of the western and eastern stocks have long been known to overlap, uncertainty surrounding stock origin makes it difficult to incorporate mixing into management strategy evaluation frameworks^49,77^. The composition of catches in the Central and Eastern Atlantic is critical; here the contribution of western origin individuals is highly variable and at times considerable^34,49^. Due to its smaller size relative to the eastern stock of ABFT, the western stock is particularly vulnerable to over-exploitation within mixing areas. Similarly, error and uncertainty in estimates of stock composition has a particularly strong influence on the assessment of western ABFT^78^. The integrated method of stock discrimination offers a highly accurate tool for discriminating between ABFT of western and eastern origin that resolves some disagreement between genetic and stable isotope based assignments and can provide reliable estimates of stock composition. The results provide further evidence that there is substantial movement of western ABFT across the 45° W management boundary which should be incorporated into the assessments. By taking an holistic approach to stock discrimination of ABFT, this study has identified contingents of ABFT within the mixing areas that are dissimilar to the majority of adults spawning in the Gulf of Mexico and the Mediterranean Sea and cannot be assigned to either using the integrated method (Med-like genetics and GoM-like chemistry or GoM-like genetics and Med-like chemistry). These fish may belong to another spawning unit or they may originate from the main spawning areas but follow a divergent early migration pathway. Future investigations of spawning and migratory contingents in ABFT should incorporate both genotypic and phenotypic information to build a more complete picture of stock complexity.

## Supplementary information

Supplementary Legends.

Supplementary Table S1.

Supplementary Table S2.

## Data Availability

Stable isotope and genetic data for the adult baselines are included in the supplementary information section (Tables S1 and S2). Other datasets analysed in the study are available from the corresponding author on reasonable request.
