# Supplementary material for: Combining genetic markers with stable isotopes in otoliths reveals complexity in the stock structure of Atlantic bluefin tuna (Thunnus thynnus)
Source: Sci Rep. 2020 Sep 7;10:14675. doi: 10.1038/s41598-020-71355-6 (PMC7477220; doi:10.1038/s41598-020-71355-6)
Supplement: Supplementary file 1 — Supplementary Legends. [file 41598_2020_71355_MOESM1_ESM.docx]

**Supplementary information (provided as separate attachments):**

Table S1: Otolith core stable isotope data for the adult chemistry baseline samples

Table S2: Otolith core stable isotope (δ^18^O and δ^13^C ) and genetic data (four SNP markers: RAD2, RAD26, RAD35 and RAD213) for the adult combined baseline samples.
